# Ponte Miocárdica: Amiga, Inimiga ou Ambas?

**DOI:** 10.36660/abc.20230426

**Published:** 2023-08-10

**Authors:** Biljana Parapid, Vladimir I. Kanjuh

**Affiliations:** 1 Centro Clínico Universitário da Sérvia Faculdade de Medicina Universidade de Belgrado Belgrado Sérvia Divisão de Cardiologia do Centro Clínico Universitário da Sérvia, Faculdade de Medicina da Universidade de Belgrado, Belgrado – Sérvia; 2 Academia Sérvia de Ciências e Artes Belgrado Sérvia Academia Sérvia de Ciências e Artes, Belgrado – Sérvia

**Keywords:** Ponte Miocárdica, Angina Pectoris, Insuficiência Cardíaca, Transposição das Grandes Artérias, Hamartoma

Mencionada pela primeira vez em 1737 por Reyman como achado de autópsia,^1^ descrita em 1922 por Crainicianu,^2^ nomeada por Polacek^3^ em 1956 e angiograficamente demonstrada por Portmann e Iwig^4^ em 1960, a ponte miocárdica abrange a porção tunelizada de uma artéria coronária, enquanto a banda muscular que a envolve é denominada ponte miocárdica (PM). Uma ponte miocárdica pode permanecer silenciosa por toda a vida ou causar uma miríade de sintomas clínicos – desde angina,^5^ e isquemia,^6^ passando por síncope^7^ com arritmia^7 , 8^ e insuficiência cardíaca^9^ até morte súbita^10 , 11^ – entretanto, nem as crianças são poupadas do quadro da cardiomiopatia hipertrófica (CMPH).^7 , 12^

Do ponto de vista anatomopatológico, três questões principais permanecem sem resposta: (1) As diferenças étnicas tornam alguns cantos do mundo mais suscetíveis a nascer com essa anomalia congênita da artéria coronária? (2) Quais são as implicações de PMs únicas ou múltiplas de vasos únicos ou múltiplos, mesmo na ausência de CMPH? Além disso, (3) Qual é a carga aterosclerótica real do segmento tunelizado e periponte?

Nesta edição dos Archivos Brasileiros de Cardiologia, o artigo original de Lucena et al.,^13^ nos dá um vislumbre das 3 questões não respondidas em sua amostra local do estado brasileiro do Ceará, um estado multiétnico *per se* , onde os autores identificaram presença dominante de PM no sistema coronariano esquerdo com maior índice muscular [IMM= comprimento da PM X espessura da PM (mm)] da PM do que o encontrado em outros ramos acometidos implicando pior prognóstico.

Globalmente, esses achados implicam que a diversidade populacional e a inclusão nas análises sempre importam, pois os números continuam variando tanto na autópsia.^14 - 17^ quanto na angiografia.^18 - 21^ As PMs tendem a se localizar mais no sistema esquerdo, e Loukas et al.,^15^ descobriram que é dependente da dominância da artéria coronária em sua amostra; no entanto, PMs sobre a artéria coronária direita também são descritas,^22^ enquanto presença concomitante com valva aórtica quadricúspide,^23^ transposição de grandes artérias,^24^ hamartoma,^25^ Takotsubo e dissecção espontânea da artéria coronária^26 , 27^ foram recentemente relatados.

Como as ferramentas de diagnóstico avançaram substancialmente nas últimas duas décadas,^28^ a avaliação de pacientes PM que se apresentaram a um cardiologista permanece em função da logística local^17 , 20 , 29 - 32^ e da experiência em manejo de longo prazo. No entanto, antes de chegarmos à decisão de tratamento, o quando, o onde e como as PMs se tornam amigas, inimigas ou ambas, de nossos pacientes em função de seu papel na aterogênese permanece o ponto central.

Enquanto Matta et al.,^33^ e Darabant et al.,^21^ consideram a presença de PM associada à redução do risco aterosclerótico e até mesmo protetora em seguimento de 10 anos, respectivamente, McLaughlin et al.,^30^ e Lu et al.,^31^ mostraram independentemente que a aterosclerose ligada à PM é mediada por mecanismos complexos mediados por adipogênese e angiogênese. O achado prevalente de aterosclerose proximal a PM é confirmado por numerosos autores,^6 , 14 , 19 , 34 - 36^ confirmando diferentes influências de longo prazo nos resultados.^20 , 37^ No entanto, com um relatório recente perturbador de PM sendo um preditor independente de arritmia fatal em pacientes com HCMP.^38^ Ao mesmo tempo, naqueles livres de CMPH, Zhang et al.,^36^ encontraram correlação positiva entre compressão sistólica e eventos coronarianos adversos maiores e aterosclerose proximal à PM.

O plano de manejo privilegia o médico, incluindo a reabilitação física,^28^ considerando que a intervenção percutânea permanece discutível mesmo nos centros mais experientes devido a inúmeras complicações de curto e longo prazo, então a inicialmente temida abertura cirúrgica oferece a solução mais promissora e permanente.^39 - 44^

Finalmente, como as disparidades de sexo nos cuidados continuam sendo a maldição da cardiologia em todo o mundo e a atual falta de diferenças de sexo em relatórios de PMs onde os homens dominam, a questão lógica parece ser se isso se deve à tradicional falta de inclusão de mulheres como pacientes em ensaios e registros que continuaram apesar da pandemia de SARS-CoV2,46,47^45^ ou é realmente uma questão de puro golpe de sorte genético que as mulheres sejam, de fato, menos afetadas. Com o objetivo de mitigar esse papel do destino e como o conceito de centros cardíacos para mulheres foi promovido globalmente,^46 , 47^ o grupo sérvio^48^ tem uma clínica dedicada – dentro de seu programa de coração para mulheres – para mulheres diagnosticadas com PM, entre outras anomalias da artéria coronária que endossam outras atividades de defesa do Centro do Coração Feminino “Dr. Nanette Kass Wenger” e ajudam a construir registros internacionais com o objetivo de preencher a lacuna do sexo – disparidades nos cuidados cardiovasculares. Se transformar inimigos em amigos-inimigos e amigos-inimigos em amigos em tempo integral continua sendo um desafio diário para todos os seres vivos na Terra, pelo menos não deveria ser o caso com diagnósticos facilmente detectáveis e suas complicações previsíveis e evitáveis com risco de vida no século XXI.
