# Supplementary material for: Ponte Miocárdica: Amiga, Inimiga ou Ambas?
Source: Arq Bras Cardiol. 2023 Aug 10;120(7):e20230426. [Article in Portuguese] doi: 10.36660/abc.20230426 (PMC10519348; doi:10.36660/abc.20230426)
Supplement: Translation

# Translation: Myocardial Bridge: Friend, Enemy, or Frenemy?

**Authors:** Biljana Parapid, Vladimir I. Kanjuh

**Affiliations:** 1 Sérvia Divisão de Cardiologia do Centro Clínico Universitário da Sérvia, Faculdade de Medicina da Universidade de Belgrado, Belgrado – Sérvia; 2 Sérvia Academia Sérvia de Ciências e Artes, Belgrado – Sérvia

**Keywords:** Myocardial Bridging, Angina Pectoris, Heart Failure, Arterial Switch Operation, Hamartoma

First mentioned in 1737 by Reyman as an autopsy finding,^1^ described in 1922 by Crainicianu,^2^ named by Polacek^3^ in 1956, and angiographically shown by Portmann and Iwig^4^ in 1960, myocardial bridging encompasses the tunneled portion of a coronary artery, while the muscle band enveloping it is termed the myocardial bridge (MB). A myocardial bridge may remain silent lifelong or cause a myriad of clinical symptoms – from angina^5^ and ischemia,^6^ via syncope^7^ with arrhythmia^7 , 8^ and heart failure^9^ up to sudden death^10 , 11^ – however, not even children are spared irrelevant of the setting of the hypertrophic cardiomyopathy (HCMP).^7 , 12^

From the mainly pathoanatomical standpoint, three main questions remain unanswered: (1) do ethnic differences render some corners of the world more susceptible to being born with this congenital coronary artery anomaly? (2) what are the implications of single or multiple MBs of single or multiple vessels even in the absence of HCMP? Furthermore, (3) what is the real atherosclerotic burden of the tunneled and peri-bridged segment?

In this issue of the *Archivos Brasileiros de Cardiologia* , the original paper of Lucena et al.^13^ gives us a glimpse into the 3 unanswered questions in their local sample of the Brazilian state of Ceará, a multi-ethnic one *per se* , where authors identified dominant presence of MBs in the left coronary system with a larger muscle index [MMI= MB’s length X MB’s thickness (mm)] of the MB than one found in other affected branches implying worse prognosis.

Globally, these findings imply that population diversity and inclusion in analyses always matter, as numbers keep varying both on autopsy^14 - 17^ and angiogram.^18 - 21^ MBs tend to be localized more in the left system, and Loukas et al.^15^ found it to be coronary artery dominance-dependent in their sample; however, MBs over the right coronary artery are also described,^22^ while concomitant presence with a quadricuspid aortic valve,^23^ transposition of great arteries,^24^ hamartoma,^25^ Takotsubo and spontaneous coronary artery dissection^26 , 27^ have recently been reported.

As diagnostic tools have advanced substantially over the past two decades,^28^ evaluation of MB-patients who presented to a cardiologist remains in the function of local logistics^17 , 20 , 29 - 32^ and experience in long-term management. However, before we reach the management decision, the when, the where, and the how MBs turn into a friend, enemy, or frenemy of our patients in the function of their role in atherogenesis remains the pivotal point.

While Matta et al.^33^ and Darabant et al.^21^ find the presence of MB to be associated with atherosclerotic risk reduction and even protective in a 10-year follow-up, respectively, McLaughlin et al.^30^ and Lu et al.^31^ independently showed MB-linked atherosclerosis to be mediated via complex adipogenesis and angiogenesis-mediated mechanisms. The prevalent finding of proximal to MB atherosclerosis is confirmed by numerous authors,^6 , 14 , 19 , 34 - 36^ confirming different long-term influences on outcomes.^20 , 37^ However, with one disturbing recent report of MB being an independent predictor of fatal arrhythmia in patients with HCMP.^38^ At the same time, in those free of HCMP, Zhang et al.^36^ found a positive correlation between systolic compression and major adverse coronary events and proximal to MB atherosclerosis.

The management plan favors medical one, including physical rehabilitation,^28^ whereas the percutaneous intervention remains debatable even in most experienced centers due to numerous short- and long-term complications, so the initially dreaded surgical unroofing gives the most promising and permanent solution.^39 - 44^

Finally, as sex disparities in care remain the bane of cardiology worldwide and the current lack of sex differences in reports of MBs where men dominate, the logical question seems to be whether it is due to the traditional lack of inclusion of women as patients in trials and registries that continued despite the SARS-CoV2 pandemic^45^ or is it truly a matter of pure genetic *coup de chance* that women are, indeed, less affected. Aiming to mitigate that role of fate and as the concept of heart centers for women has been promoted globally,^46 , 47^ the Serbian group^48^ has a dedicated clinic – within its women’s heart program – for women diagnosed with an MB, among other coronary artery anomalies that endorse other “Dr. Nanette Kass Wenger” Women’s Heart Center advocacy activities and helps build international registries aiming to fill the gap of sex-disparities in cardiovascular care. If turning enemies into frenemies and frenemies into full-time friends remains a daily challenge for every living being on Earth, then at least it should not be the case with easily detectable diagnoses and their foreseeable and preventable life-threatening complications in the 21^st^century.
